# Violence Against Black Lives Matter Protestors: a Review

**DOI:** 10.1007/s40719-022-00228-2

**Published:** 2022-05-27

**Authors:** Mihir J. Chaudhary, Joseph Richardson

**Affiliations:** 1 Department of Surgery, University of California San Francisco – East Bay, 1411 E 31st Street, Oakland, CA 94602 USA; 2 University of Maryland at College Park, College Park, MD USA

**Keywords:** Black Lives Matter, Political violence, Police violence, Right-wing violence, Protestor injuries, Racial justice

## Abstract

**Purpose of Review:**

The Black Lives Matter (BLM) protests against racialized police violence represents the most prolific mass social movement in modern times. It has been met by sustained and repressive violence by state authorities and right-wing groups. This review seeks to synthesize existing scholarly, journalistic, case report, and crowd sourced data on violence directed against BLM protestors.

**Recent Findings:**

Data from various sources suggests that police disproportionately target BLM protests for violent intervention. There is also mounting evidence of organized and vigilante right-wing violence targeting BLM protestors. While police frequently use chemical irritants and projectiles, right-wing protestors often use car ramming to bluntly injure protestors. The true scale and nature of injuries affecting BLM protestors remains unknown though injuries resulting in the need for intensive care, operative intervention, permanent morbidity, and mortality have been reported.

**Summary:**

State and conservative civilian violence against BLM protestors represents an ongoing threat to the right to organize and publicly dissent. The use of force manifested by police and right-wing groups against BLM protestors resulted in significant injury and mortality.

## Introduction

During the summer of 2020, following police killings of Black Americans, the USA saw perhaps the most widespread protest movement in its history [1]. This was accompanied by an equally historic repression of dissent by all levels of state authority [2]. Even prior to the massive Black Lives Matter (BLM) protests, police departments in the 20 largest cities in the USA were failing to meet basic international human rights standards governing the use of lethal force [3]. During the summer of 2020, widespread incidents of state mistreatment of protestors were broadly documented throughout the news and social media. However, there remains a lack of publicly mandated or otherwise organized data collection on state violence against civilians in the USA. None of the 18,000 municipal police departments across the country is under any legal obligation to report officer-related shootings or violence to federal databases [4]. In addition, although over 90% of law enforcement departments voluntarily report violent crimes to the FBI through the Uniform Crime Reports (UCR), there is no specific category to document the use of force by police officers or other state agents. Indeed, only 4% (750 of 18,000) of law enforcement agencies reported police-involved shootings to the FBI in 2012 [5]. Much of this data is currently collected and disseminated by third parties through compilations of journalistic reports [6].

In addition to state violence against BLM protestors, the summer of 2020 and beyond also saw a precipitous rise in violence by right-wing militias and counter-protestors. Similar to police violence reporting, there is no legal obligation for law enforcement to report hate crimes or politically directed violence [7]. The FBI has a voluntary reporting system for state authorities to report hate crimes, which is limited by its reliance on the subjective assessment of law enforcement.

The purpose of this review is to (a) provide a historical context to state repression of political actors in the USA, (b) review the existing scholarly, journalistic, and civilian-collected data describing state and far-right militia and vigilante violence against BLM organizers and protestors starting in the summer of 2020, and (c) provide a framework for collecting data on state violence against protestors. Importantly, given the existing limitations in data collection described above and the relative opacity of modern municipal police departments, this review combines descriptive analysis with a review of existing, though incomplete, data sources on violence targeting BLM protestors. A primary goal of this review is to capture the scale and nature of state violence and mistreatment of political actors in the USA with a special focus on the events of the summer of 2020.

## Contextualizing State Violence Against Political Actors in the USA

Violence has been central to US state building since its inception and long before the BLM movement. Much historical research has described how the incipient US state explicitly sponsored White vigilante violence to occupy indigenous lands. For example, as White settlers sought to occupy indigenous lands, the US government offered material rewards for the scalps of those it sought to displace [8]. Anti-Black violence displays similar themes. For instance, in the Jim Crow South, White vigilante mobs would incite violence that would enable police or state intervention to maintain racial control [9]. The Tulsa massacre — with the explicit deputization and arming of White vigilantes by the city — is one such example [9]. In this way, the US state extended its hegemony in close partnership with White settlers. Whether the colonization of the Eastern coast or the extension of the US state over the Western frontier, this reciprocal relationship has held.

Violence against BLM protestors exists in a long historical arc of state repression of internal political challengers to the US state and capitalism [10]. For example, the Black Panther Party (BPP) founded in Oakland, California in 1966 emerged initially to resist the police state and organized amongst highly policed, working class Black communities in major US cities. The BPP directly monitored police with their own legally armed patrols (“copwatching”) and developed a network of free health clinics, food aid, and education programs [11, 12]. As the BPP grew, the US state deepened their surveillance and repression of the movement. This culminated in the FBI infiltration of the BPP in Chicago and execution of its chapter leader Fred Hampton just as the BPP was forming a multi-racial working class social movement [13]. In contrast to contemporary tensions between BLM protestors and White conservatives, the Chicago BPP, under Fred Hampton, politically united with groups that could easily have splintered into right-wing militias — such as the Young Patriots, an organization of working class White southerners in Chicago [14]. Civil right activists also experienced state repression in forms analogous to BLM activists. During the 1960s, television media was becoming ubiquitous and revealed the blatant use of violence against those demanding racial justice. For example, television captured images of the Ku Klux Klan attacking civil rights activists in Alabama during their 1961 Freedom Rides to promote desegregation. In this particular example, Alabama police, despite knowing the Ku Klux Klan threat to the Freedom Riders, failed to prevent their violent attack. This galvanized broad White support and delegitimized many state institutions [15]. In contrast to the more unilateral state violence and infiltration of the BPP, this episode demonstrates the connection between state and White vigilante violence, a theme that we will see predominate in violence against BLM protestors. Unlike the impact of television during the civil rights era, as time progressed with the BLM protests, a well-organized right-wing establishment co-opted the media narrative to depict BLM as violent and thus erode initial White support for the movement [16].

## State Violence and the Black Lives Matter Movement

On June 4, 2020, Martin Gugino, a 75 year-old peace activist with the Catholic Worker Movement, was in attendance at a nonviolent Black Lives Matter demonstration in Niagara Square Buffalo, New York. During the demonstration he was walking towards approaching police and was first met with a forceful baton to his chest from one officer followed immediately by a push from another officer. Mr. Gugino stumbled backwards, fell and struck his head on the pavement. He lay motionless, bleeding from his ear as several officers walked past. Mr. Gugino suffered a skull fracture and subdural hemorrhage requiring a two-week intensive care unit admission [17].

Gugino is one of the many BLM protestors and organizers who have faced direct physical harm secondary to police action during the summer of 2020. Given the lack of aggregated data collection, the full scale of police-related physical injury of BLM protestors is difficult to capture in its entirety. However, this part of the review will delve into what is known — the nature and recorded scale of police mistreatment of protestors — and its implications for the future of civic action in the USA.

In the absence of official state or legal data, the Armed Conflict Location and Event Data project (ACLED) exists as one of the most reliable source of data to track protest-related political violence in the USA during the summer of 2020 [2]. The ACLED is an independent non-profit data collaborative combining the resources and expertise of academic institutions, governmental organizations, and local partners to create a publicly available disaggregated data set on political violence around the world. While ACLED has historically focused its data gathering in the Middle East, Asia, Europe, and Latin America, it began to focus on the USA following the rise in political violence during BLM protest activity in the summer of 2020.

The ACLED collects data by coding over 2800 sources of data including local, state, and national media to identify political violence. In addition to media reports, the ACLED also collects information from data aggregators such as the Crowd Counting Consortium to tabulate political violence [18]. An inherent limitation to the ACLED is its over-reliance on media sources, which may exhibit bias in the type of events captured and may miss a number of events involving fewer people or in more remote locations. Nonetheless, the ACLED provides the single largest collection of data on BLM protests in 2020, recording 10,330 BLM demonstrations across 50 states (including Washington, DC) in 2730 distinct locations [19].

The ACLED dataset describing political violence during the summer of 2020 — the “US Crisis Monitor” — found that 9% of BLM demonstrations were met by some form of state intervention (including use of physical force as well as other interventions) compared to 4% of right-wing demonstrations and 3% of all other demonstrations in the same year. This targeting of BLM protestors is despite the fact that 94% of BLM demonstrations were non-violent and non-destructive compared to 86% of right-wing protests. Importantly state intervention at BLM protests was typically more violent than those at right-wing demonstrations. Indeed 51% of BLM protests were met with physical force (this included use of less-lethal projectiles like tear gas or rubber bullets to beating protestors with batons) compared to 33% of right-wing demonstrations and 26% of other demonstrations [19]. The ACLED codes right-wing demonstrations as encompassing a wide range of interrelated ideological stances or focus areas that include pro-Trump, Republican, pro-police, ‘Stop the Steal’ events or direct counter-protests to BLM demonstrations.

ACLED data also re-affirms the historically consistent reciprocity between the US state and right-wing militia actors. Similar to the collusive episode during the CIvil Rights era, described earlier, between Alabama state authorities and the Ku Klux Klan, there have been important interplays between the US state authorities and White right-wing militia groups like the Proud Boys. Indeed in response to Trump’s command to such extra-legal entities to “stand back, stand by” there were multiple subsequent episodes of Proud Boys initiated street-fighting during demonstrations in North Carolina, New York, California and Washington DC [20]. There are also striking examples of unaffiliated or loosely affiliated White vigilantes directly responding to perceived calls to action from the state. For example, Kyle Rittenhouse, a right-wing White vigilante, killed two BLM protestors following a perceived muster call from the National Guard [19]. He viewed the presence of the National Guard as an indication to attack. In general, the presence of right-wing demonstrators or militias tends to increase episodic violence during BLM protests. According to the ACLED database at least 38 distinctly named far-right groups have directly engaged with BLM protestors and 26% of these events have resulted in violence. Again the alignment between political conservatism and the police state is clear in that only 2% of ‘Back the Blue’ pro-police demonstrations experienced state intervention compared to nearly 10% of despite BLM protests being equally, if not more, peaceful [20].Given the media bias present in reporting on BLM events it is highly likely that the ACLED data set misses many episodes of state violence against protestors that are sporadic or occur in less urban areas. Later this review will discuss the myriad community and citizen driven data collection systems that have emerged to fill in the gaps in data collection on violence against BLM protestors.

### Violence Against Protestors and the Militarization of American Police

Many of the technologies and vehicles deployed during the mass protests in the summer of 2020 and beyond reflect the growing militarization of United States municipal police [21]. Additionally, though again precise data is scarce, many of the protests in major American cities received a coordinated response between local, state and national law enforcement. The National Guard and state-level police forces were a regular accompaniment to city police during the response to BLM protests in major cities [22].

Some specific examples of police militarization during BLM protests include the use of Mine-Resistant Ambush Protected or MRAP armored vehicles typically designed for military conflict zones. Such vehicles have been deployed around the country both as an intimidation and crowd control technique [22].

Additionally, human rights organizations document that police utilized a basic, recurring set of techniques to escalate protests and make violent confrontation more likely such as “trap and detain” or “kettling” whereby militarized vehicles are employed to encircle protestors. These situations often lead to use of physical force and create pretexts for mass arrests [22].

## Far-Right Response to BLM protests

Importantly, as indicated earlier, in addition to state authorities, far-right civilian militias have engaged in violence directly against BLM protestors and often complement or synergize with police-based tactics to stifle dissent. Indeed the scale of counter-demonstrations to BLM protests has been substantial with the ACLED recording over 750 distinct counter-demonstrations directly in response to BLM demonstrations between January 2020 and April 2021 [19]. Within the “far-right” exists a myriad of similarly ideologically aligned actors. Here we specifically explore violence against BLM protestors stemming from individuals and militias. These are defined in turn and their approach to violence against BLM protestors described.

### Individuals

Individuals ideologically connected to far-right movements in virtual spaces often acted independently to contest various BLM protests. One such example, mentioned earlier was Kyle Rittenhouse’s response to the Kenosha Guard muster call and his subsequent killing of two BLM protestors. In fact armed individuals associated with right-wing movements may see themselves as auxiliary to police or military and respond to their perceived calls. Similar to Rittenhouse, Steven Ray Baca responded to a muster call by the New Mexico Civil Guard. During a symbolic struggle over the statue of Spanish conquistador Juan de Oñate, Baca shot and wounded a BLM protestor. Both Baca and Rittenhouse demonstrated ties to the conservative political establishment. Baca ran for city council as a board member of the Albuquerque Tea Party [23]. In the case of Rittenhouse, the Republican establishment sought to frame him as a defender of property owners and small business owners. [24] Right-wing actors actively mobilized a narrative to justify violence against BLM by making the movement appear dangerous to everyday civilians.

### Militias

Far-right militias constitute organized and armed citizens that essentially operate as a paramilitary force [24]. In the modern era these groups often view themselves as operating under the implicit leadership of major conservative political leaders such as the relationship between the Proud Boys and former President Trump. These groups exist on a spectrum of actually constituting organized militia groups with rank ordered units (the Fraternal Order of the Alt-Knights (FOAK)) to more broadly violence-espousing, militant groups such as the Groypers. Some of these groups have significant numbers of former police officers and military personnel as members (the Oath Keepers is a notable example) [25].

Such far-right militias and militant groups have displayed an increasing presence at BLM protests over time. Indeed there have been at least 38 distinctly named far right militia or militant social groups that have directly confronted BLM protests. According to ACLED data the most common organization involved has been the Proud Boys. This group has countered almost 50 demonstrations and in over 36% of these engaged in violence or destructive acts [19].

Importantly, the different far right militias or militant groups are aligned ideologically in their opposition to BLM though they often have different strategies of inciting violence against protestors. For example, the Boogaloo Boys — an extremist far-right group aiming to trigger widespread violence and societal collapse — has located itself in BLM protests as provocateurs, hoping to catalyze large-scale violence. Interestingly, the Boogaloo Boys, distinct from other groups, is not monolithically a White supremacist organization and is knit together by their shared anti-governmental ideology and a desire for the collapse of society [26]. The complexity of their relationship with BLM is evinced by the fact that they have been in direct opposition to BLM activists in 40% of recorded protests and actually expressed support in 60% of protests. In 10% of protests where Boogaloo Boys are present they have sought to incite larger scale violence [19].

A key and common strategic response of these groups has been to establish an armed presence at BLM protests. At least 25 distinctly named groups have brought visibly armed members to BLM protests often in the self-proclaimed role of containing the violent or destructive behaviors of protestors, again indicating their self-perception as an auxiliary to state violence [19].

## Nature of Injuries Sustained by BLM Protestors

In addition to the scale of violence against BLM protestors during the summer of 2020, it is important to note the type of injuries sustained. This next section delves deeper into the discrete public health and clinical impact of the violence inflicted by state authorities and right-wing counter-protestors against BLM activists.

The racial justice protests of 2020 revealed law enforcement reliance on a particular set of technologies to quell protest. One particularly widespread technology used were chemical irritants, such as tear gas, to disperse gathered crowds. A systematic review of studies on the health impacts of tear gas spanning 29 studies (including 7 from the United States) across 11 countries and 5131 individuals found a total of 9261 injuries and that two people died related to chemical irritant complications and 58 suffered permanent disability [27]. Out of the 58 people suffering permanent debility 18 were related to projectile injury from a tear gas canister. This ranged from permanent vision loss secondary to globe ruptures to limb amputations and traumatic brain injury leading to a permanent vegetative state. The remaining 40 permanent disabilities were chronic respiratory, dermatological and psychiatric in nature. A far greater number (n = 6878) suffered mild injury, mostly dermatological, ocular and cardiopulmonary [27].

Significantly, the most serious tear gas related injuries stem from the projectile canister rather than the chemical irritant. Indeed state authorities made widespread use of so-called “less than lethal” or “non-lethal” projectiles against BLM protestors that included rubber bullets, pepper balls, beanbag rounds and propelled tear gas canisters. A systematic review spanning 26 articles (including three from the United States) from 1990 to 2017 compiled injury data on 1984 individuals of which 53 died from such projectiles [28]. Half of these deaths were related to head and neck injury — a body area that most US municipal police crowd control guidelines explicitly forbid targeting. [29] About a quarter of deaths were related to chest and abdominal trauma. Of those surviving their injuries (*n* = 1931), 300 people suffered permanent disability, most commonly vision loss and abdominal injuries requiring splenectomy or colostomies. Two people suffered limb amputation. This study also found that the muzzle velocity of the “less than lethal” projectiles matched that of lethal ammunition [28].

The exact scale of penetrating and chemical irritant related morbidity and mortality secondary to state action during the protests of the summer of 2020 remains unknown. However, case series during the BLM protests give a sense of the type of injuries these police technologies can inflict. One particular report from Austin, Texas following two days of BLM protests describes the impact of beanbag and rubber bullet munitions on 19 patients treated at the local level 1 trauma center. [29] Eight of the patients presenting after injury required admission and seven required operative intervention. Four of the patients suffered intracranial hemorrhage with one requiring a decompressive craniectomy for increased intracranial pressure and another patient with a depressed frontal bone fracture requiring a craniotomy and cranioplasty. Other patients required fixation of facial fractures, removal of foreign body retained in the chest, splinting for finger fractures and repairs of lacerations in the scalp or extremity.

Another larger sample study of “less than lethal” munitions injuries during the summer of 2020 from Minnesota performed a retrospective review between May 26 and June 15, 2020 of all patients possibly injured during protests [30]. During this study they identified a total of 89 individuals sustaining some sort of injury. Of these 45 individuals (51%) were injured by projectiles, 32 (36%) were injured by chemical irritants and 12 (13%) by a combination of chemical irritants and projectiles. A total of 16 (18%) individuals suffered a traumatic brain injury and 7 (8%) required surgical intervention for their injuries. The operative interventions included two explorations for a ruptured globe, fixation for a clavicular facture, elevation of depressed skull fracture, left testicular debridement, enucleation of eye, and debridement and repair of extremity laceration. Similar to other studies on projectile injuries in such settings, a high rate of injuries, 23 of 57 (40%) occurred in the head, neck or face.

A final case series of penetrating injury secondary to police projectiles reviewed 14 patients presenting to a level 1 trauma center in Los Angeles over a 48-h period during BLM protests [31]. This series found one patient to suffer multiple intracranial hemorrhages requiring intensive care. Eight patients suffered facial fractures and lacerations, most requiring complex wound care with one requiring surgical fixation of a mandible fracture. One patient suffered an orbital blowout fracture and lost their vision. Three patients suffered superficial lacerations requiring suture repair. One patient suffered a testicular rupture requiring surgical repair. One patient suffered an open ulnar fracture requiring surgical repair. Of note this study and the prior two studies constitute the total identified clinical case series data on injuries sustained by BLM protestors, revealing the major need for data collection. They also provide methodological proof of concept that trauma centers can identify protestors injured by police and state authorities.

Beyond the documented clinical case series of projectile injuries, blunt injury by car rammings has also been an increasingly prevalent form of violence directed against BLM protestors. Car rammings occurred eight times more frequently at BLM protests compared to other demonstrations and were exclusively initiated by right-wing individuals or groups [2]. There have been at least fifty recorded incidents of right-wing use of vehicles to bluntly injury or kill BLM protestors since the start of protest activity in June 2020 [32]. There have been at least fifty additional episodes of car rammings with undetermined intent. Twenty people faced prosecution for utilizing their vehicles as a weapon including the state leader of the Virginia Ku Klux Klan [33]. There are, as of yet, no case series or retrospective data delineating the nature and severity of injuries resultant from such car rammings. Importantly, in another example of the alignment of the state with far-right violence, is the passage of laws in Republican-led states providing legal protections for drivers who hit protestors [19].

It is important to note that while the BLM protests generally become more peaceful over time the public perception was that they were becoming more violent. As described earlier, this is in marked contrast to how the public perceived the civil rights movement which gained more public and more White support as televisions streamed the undeniable state violence against peaceful protestors. Such a difference points to the relative importance of curated social media in shaping today’s public views. Indeed according to Pew surveys the BLM protests became *more* peaceful over time. Between May 25 and June 4, 2020, 89% of protest demonstrations were non-violent while later between June and September 2020 more than 96% of BLM associated protests were non-violent [16]. Nonetheless, the right-wing political establishment shaped public opinion such that more Americans disapproved of BLM as time went on. In the immediate aftermath of Floyd’s killing in June 2020, 67% of Americans supported the movement. By September 2020 this number had dropped to 55% of adults. The drop in support was largely limited to White Americans of whom 60% initially supported the movement with a drop down to less than half a few months later [16].

## Approaches to Collecting Data on State Violence Against Protestors

As evidenced by this review the ability to understand the scale and nature of state violence against political actors is limited by the sources of data available. The particular challenge of collecting data on state violence is that the government, which usually has the resources and institutional reach to collect the necessary information for a data set, is also the very entity being interrogated. To come around this problem, researchers and advocates have long proposed alternate methods to improve data collection on officer-involved violence such as equipping trauma centers to gather this information directly from patients. Nonetheless systematic approaches or legal mandates to acquire this information remain notably absent.

The current era of public organizing has been accompanied by dramatic episodes of violent repression that highlights the importance of data collection and dissemination. In response, activist individuals and organizations have deployed numerous extra-institutional approaches to data gathering. Considerations for collecting data on political violence against protestors, a review of current data collection efforts and ways to politicize such data are described further.

While medical professionals and healthcare systems should continue collecting data on state violence as it impacts their patients, episodic violence impacting protestors requires specialized considerations:

1. Protestors may be less likely, especially in the era of COVID-19, to present to the formal healthcare system following state-sanctioned injuries

Data demonstrates a global decrease in utilization of emergency room services since the beginning of the COVID-19 pandemic [34]. This decrease in utilization likely affects injured protestors as well. Moreover, protestors are often younger and thus more likely to be uninsured, further decreasing their utilization of the healthcare system [35, 36]. Informal, volunteer “street medics” frequently serve as the first line of treatment for injured protestors [37]. Since many protestors receive street medic treatment or self-treat at home, alternate methods are required to collect injury data.

2. Protestors may be less likely to report state violence to institutional authorities

For the same reasons listed above, namely general distrust of institutional authority, even those protestors who present to a hospital-based setting may be less likely to report state-sanctioned violence. This distrust is further heightened amongst Black Americans given historical abuse by medical institutions [38]. Indeed hospitals collect patient name and other identifying information, which may limit the comfort of protestors to share contextual information about their injuries. Many hospital settings also have law enforcement presence, which may further limit what protestors may share especially if they have prior justice system involvement. Additionally, given the level of state surveillance accompanying protests, it is reasonable that organizers and activists would remain discreet. It is thus necessary to create data collection techniques that empower these groups to contribute to the task of data collection while protecting their identities.

3. Creating open source data portals allows multiple types of mistreatment to be captured

Publicly available social media data archiving over a thousand separate instances of state violence and mistreatment against protestors reveals an incredible range of events that may only be partially captured by current dataset, such as ACLED, reliance on media based reports. This includes physical violence as well as verbal abuse and arbitrary arrest. Extra-institutional data collection methodologies should be flexible to capture the entire range of state-based mistreatment of protestors.

Linked to the first two reasons described above formal trauma databases, such as the National Trauma Data Bank (NTDB), which collect comprehensive injury related data as reported directly from hospital trauma centers, may only capture a portion of protest injury data [39]. Thus most trauma researchers, in their reliance on sources such as NTDB, will be limited in their ability to study state violence against protestors. Nonetheless hospital based data collection still remains an important source of data as reflected in the prior three retrospective protest injury studies reviewed (from Austin, TX; Minneapolis, MN and Los Angeles, CA) [27, 30, 37] Other approaches, described further, can bolster hospital-based data collection.

### Current Efforts to Collect Data

Ongoing movements for racial justice as well as the movement to collect data on racialized police brutality are increasingly decentralized. Data for Black Lives is an example of efforts to collect data to bolster the movement with the mission of “using data science to create concrete and measurable change in the lives of Black people” [40]. Another, recent volunteer organizational effort to leverage data for racial equity is the COVID Tracking Project [41]. Multiple other efforts have emerged to collect data specifically focusing on widespread state violence and mistreatment against protestors. While these projects are now variably active, their techniques may hold potential for future data collection strategies:

1. Twitter archives: Twitter has been a central source of reporting and collecting data on state repression of protests as evidenced by the archive of instances amassed by T. Greg Doucette [42]. Various individuals have utilized this source of information to compile over a thousand episodes of state sanctioned violence and mistreatment of protestors. This information has been further adapted into mobile apps that identify the locations of police brutality and mistreatment of protestors and also of civilians more generally (The COVID Tracking Project).
2. The Witness Archives: An independent initiative to collect and publish incidents of police violence and mistreatment against protestors captured on video by protestors [43].
3. Geographic Information Systems (GIS) spatial mapping of police brutality: Several independent efforts have sought to synthesize social media derived data into GIS based maps highlighting the regional scale of state violence against protestors [44].
4. Raheem: Another independent portal to collect data on police brutality. This service mobilizes the narratives of those impacted by the police and translates them into various forms of political engagement including formal complaints and pursuits of justice [45].

In addition to the efforts listed above, there remains an enormous potential to widen data collection by empowering protestors to directly report state violence and mistreatment in real time. An ultimate, longer-term data goal would be to synthesize the various reporting mechanisms into a central database for research and policymaking purposes. Information about the scale and nature of political violence and mistreatment against protestors can inform policy at local, state and national levels. Specifically this data can be brought to bear on hearings or directly provided to legislators considering the impact of state and right-wing violence on communities.

Finally, synthesizing and analyzing such data is particularly important to health professionals caring for trauma patients regardless of one’s political values. As noted above the nature of injuries sustained by BLM protestors, whether due to state or right-wing actors, resulted in significant morbidity and mortality during the 2020 BLM demonstrations. Protestors often required operative intervention and critical care for the treatment of their injuries. Trauma surgeons are often the primary treating clinicians for such injuries and, as such, may be trusted with important information regarding protest related injury. Indeed recent literature has demonstrated the unique trust patients place in their surgeons [46]. This may translate into greater patient readiness to disclose state and right-wing violence. While ensuring confidentiality, such information represents an opportunity for hospital-based data collection either through the NTDB or institutional level registry. The reason why the only existing (albeit limited) clinical data on protestor injury comes from three hospital level reports may be because of patient trust and willingness to disclose the context of their injury to their treating provider. At the level of prevention, trauma surgeons and other clinicians can use their experience treating patient injuries to drive policy reforms to limit harmful crowd control technologies.

## Conclusions

This review has sought to provide a quantitative and descriptive sense of violence against BLM protestors in 2020. Importantly this violence exists in historical continuity with other challenges to US state legitimacy including explicit attempts to undermine larger scale political projects for racial justice by groups like the Black Panther Party and myriad others during the civil rights era.

The data that currently exists on violence against BLM protestors is incomplete and biased towards media reports on larger scale urban protests. It is apparent, however, that BLM protestors, relative to other demonstrators, have been disproportionately affected by state and right-wing violence. Predominant mechanisms of injury include chemical irritants, projectiles and intentional blunt automobile impact. In reviewing the damage incurred by chemical irritants and “less than lethal” projectiles it is clear these technologies can cause major injuries and even mortality. Additionally, blunt trauma in the form of car rammings, and the legal protections for those who engage in them, represent a highly morbid and often lethal technique used by right-wing actors against BLM protestors. Overall the data reviewed is limited by the inherent biases of media based reports that may be even more pronounced in the case of BLM [46, 47]. However, other data sources that aggregate individual reports from social media or provide publicly accessible platforms have emerged in the wake of the BLM protests.

There exists growing evidence that the political action of BLM protests has impacted police lethal use of force with a May 2021 study demonstrating a 15–20% decrease in police homicides in census tracts with protests between 2014 and 2019 [48]. This review demonstrates that such social change can come at grave physical cost to organizers and activists. The medical community, particularly trauma surgeons, should be aware of such violence given the tangible clinical impact they have on patients. Trauma practitioners may ultimately be the first line of data collection for such forms of violence at the hospital level. Injuries witnessed and managed by trauma surgeons can make the nature of such political violence accessible to lawmakers.
